# Towards breed formation by island model divergence in Korean cattle

**DOI:** 10.1186/s12862-015-0563-2

**Published:** 2015-12-18

**Authors:** Eva M. Strucken, Seung H. Lee, Gul W. Jang, Laercio R. Porto-Neto, Cedric Gondro

**Affiliations:** University of New England, School of Environmental and Rural Science, Armidale, Australia; Division of Animal & Dairy Science, Chungnam National University, Daejeon, Korea; Rural Development Administration, National Institute of Animal Science, Wanju, Korea; Scientific and Industrial Research Organization, Agricultural Flagship, Brisbane, Australia

**Keywords:** Hanwoo, Jeju Black, Chikso, Brindle, Effective population size, Conservation, Autochthonous breeds, Genetic diversity

## Abstract

**Background:**

The main cattle breed in Korea is the brown Hanwoo, which has been under artificial selection within a national breeding program for several decades. Varieties of the Hanwoo known as Jeju Black and Chikso were not included in the breeding program and remained isolated from the effects of recent artificial selection advancements. We analysed the Jeju Black and Chikso populations in regards to their genetic variability, state of inbreeding, as well as level of differentiation from the mainland Hanwoo population.

**Results:**

Jeju Black and Chikso were found to have small estimated effective population sizes (*N*_*e*_) of only 11 and 7, respectively. Despite a small *N*_*e*_, higher than expected heterozygosity levels were observed (0.303 and 0.306), however, lower allelic richness was found for the two island populations (1.76 and 1.77) compared to the mainland population (1.81). The increase in heterozygosity could be due to environmental disease challenges that promoted maintenance of higher genetic variability; however, no direct proof exists. Increased heterozygosity due to a first generation crossing of genetically different populations is not recorded. The differentiation between the Korean populations had *F*_*ST*_ values between 0.014 and 0.036 which is not as high as the differentiation within European beef or dairy cattle breeds (0.047–0.111). This suggests that the three populations have not separated into independent breeds.

**Conclusion:**

Results agree with an island model of speciation where the brown Hanwoo represents the ancestral breed, whilst the Jeju Black and Chikso diverge from this common ancestor, following different evolutionary trajectories. Nevertheless, differences are minor and whether Jeju Black and Chikso cattle will develop into discrete breeds or reintegrate with the main population has to be seen in the future and will largely depend on human management decisions. This offers a rare opportunity to accompany the development of new breeds but also poses challenges on how to preserve these incipient breeds and ensure their long term viability.

**Electronic supplementary material:**

The online version of this article (doi:10.1186/s12862-015-0563-2) contains supplementary material, which is available to authorized users.

## Background

Recent developments in genome-wide data collection have enabled researchers to construct world-wide patterns of ancestry and admixture in domesticated cattle [1, 2]. Whilst these studies provide an exceptional overview of diversity patterns, smaller sub-populations that are just on the verge of becoming a distinct new breed were not included. Genetic differentiation usually takes places when individuals of a population get separated and diverge from each other driven by natural selection, founder effects, genetic drift and the lack of intermixture between the populations. A classic example is Darwin’s finches with many different (sub) species inhabiting the multitude of islands in the Galapagos, each adapted to the specific environmental conditions of their island [3, 4]. A similar scenario can be found in Korea, where a large cattle population exists on the mainland (brown Hanwoo), kept for beef production purposes (carcass weight and marbling) and therefore undergoing artificial selection pressure [5], and two other cattle populations (Jeju Black and Chikso) inhabiting small areas on the mainland as well as two islands off the coast line. On the mainland, the brown Hanwoo dominate mainly due to their incorporation into the national breeding program since the 1970s. On Jeju Island in the South, black Hanwoo (Jeju Black) survived, whilst some small populations of brindle or tiger-striped Hanwoo (Chikso) can be found on the mainland and on Ulleung Island in the East. Up until now, these breeds have been treated as independent breeds, mainly due to their different coat colours (National Report on the State of Animal Genetic Resources of the Republic of Korea; 2004).

Cattle bones found on Jeju Island were most closely related to Jeju Black suggesting that the progenitors of these cattle inhabited the island already 1100–2000 years ago [6]. This time span coincides with migration routes from North China to Japan via the Korean peninsula [7]. Jeju Black were used as presents to the king and selected for their black coat colour during the Joseon Dynasty (1392–1897). In 1992, the population was on the brink of extinction and had been reduced to about only 30 animals. As a result of conservation efforts, the population of Jeju Black has increased, though current figures for population size vary widely. Most Jeju Black cattle are kept in two preservation centres on Jeju Island. In 2013 the population was designated a natural monument of Korea with the hope of drawing attention to the need of improving the lineage and disease control measures, as well as raising awareness about the historical significance of the breed (Jeju Special Self-Governing Province Community).

First records of Chikso cattle can be dated back to a picture on an ancient tomb mural from AD 357 (Domestic Animal Diversity Information System, DAD-IS, FAO). The Chikso cattle nowadays comprise about 4000 animals of which 3000 exist on the mainland and another 1000 animals on Ulleung Island.

Both Jeju Black and Chikso were classified as endangered by the National Report on the State of Animal Genetic Resources of the Republic of Korea (2004). However, exact numbers of total or effective population sizes are not recorded and studies on the genetic divergence of the breeds as well as their genetic variability and inbreeding are sparse [8–11]. Maintaining genetic diversity, especially in small populations, is of high importance to prevent a decline in health and fertility and to preserve the ability of a population to respond to environmental changes in the future [12]. Preservation of Jeju Black and Chikso cattle is of cultural value because of their ancient origins and strong links to the history of Korea.

Besides the varieties of Hanwoo within Korea, Chinese Yanbian cattle have been shown to be genetically highly similar to Hanwoo [13] but have maintained a higher level of genetic diversity possibly due to the lack of artificial selection within an organized breeding program. The Yanbian region in China has a strong Korean influence [14] and the Yanbian cattle were probably fully connected to the brown Hanwoo until the split between North and South Korea in 1953. Therefore, the Yanbian can be seen to some extend as a proxy for the original Hanwoo population prior to the implementation of the national breeding scheme.

Here, we provide important information (based on genome-wide markers) about the state of the Jeju Black and Chikso populations in regards to their genetic variability, state of inbreeding, as well as level of differentiation from the mainland Hanwoo population. Results of this work should be of value for practical decision making on how to best conserve these populations.

## Results and discussion

### Variability and Isolation

A breeding program for Korean cattle was established as early as the 1930s. In the late 70s selection was intensified and the current breeding objectives were adopted. However, this program only included the mainland brown Hanwoo cattle populations. The Jeju Black and Chikso cattle populations were not part of the breeding program and as a result their population sizes decreased. The actual population sizes are unknown and reports differ widely between 30 animals to 3579 animals for the Jeju Black (Domestic Animal Diversity Information Service (DAD-IS), Jeju Special Self-Governing Province Community, [15]), and around a total of 4000 animals for the Chikso. Despite our sampling of the Chikso from the mainland, we will refer to the Jeju Black and Chikso populations from now on as island populations, due to their selective differences and isolation from the brown Hanwoo. The mainland brown Hanwoo population in comparison comprised 1,239,380 individuals in 2003 (Domestic Animal Diversity Information Service (DAD-IS)) and has been on the rise with reported 3 M individuals [16]. Population size, and more specifically the effective population size (*N*_*e*_), are strongly associated with the genetic variability and the ability of a population to react to environmental changes. To get a better understanding of the size and structure of the Korean cattle populations we estimated *N*_*e*_ based on the linkage disequilibrium (*r*^*2*^) between the genome-wide markers. The effective population sizes for Jeju Black and Chikso were 11 and 7, respectively, whilst it was 97 for the mainland Hanwoo (Table 1). The effective population size for Hanwoo is in concordance with previous estimates from Lee et al. [8].

**Table 1:** Population metrics of 10 cattle breeds including effective population sizes (*N*_*e*_), genetic variance, allelic richness, and inbreeding measures (±sd)

Breed	N	*N*_*e*_	MAF	Het obs	Het exp	# Fixed markers	*A*_*R*_	*pA*_*R*_	*F*_*IS*_	G diag	G off-diag
Hanwoo	40	97 ± 31	0.23 ± 0.15	0.312 ± 0.17	0.309^†^	1321	1.81 ± 0.28^a^	0.0003 ± 0.006	0.003	0.97	0.080
Jeju Black	20	11 ± 2	0.22 ± 0.16	0.303 ± 0.19	0.292***	3367	1.77 ± 0.34	0.0003 ± 0.005	−0.013	0.99	0.150
Chikso	20	7 ± 2	0.21 ± 0.16	0.306 ± 0.20	0.287***	4023	1.76 ± 0.35	0.0002 ± 0.004	−0.039	0.97	0.162
Yanbian	39	227 ± 67	0.24 ± 0.15	0.326 ± 0.16	0.324	861	1.84 ± 0.25	0.0004 ± 0.006	0.009	0.98	0.037
Holstein	19	14 ± 4	0.24 ± 0.15	0.329 ± 0.19	0.318***	3018	1.81 ± 0.32^a^	0.0027 ± 0.040	−0.010	1.29	0.368
Brown Swiss	6	15 ± 15	0.20 ± 0.17	0.291 ± 0.25	0.262***	8115	1.73 ± 0.44	0.0004 ± 0.009	−0.018	1.26	0.459
Limousine	13	77 ± 45	0.24 ± 0.15	0.332 ± 0.20	0.318***	2797	1.83 ± 0.30^b^	0.0006 ± 0.011	−0.003	1.16	0.226
Hereford	15	26 ± 7	0.24 ± 0.15	0.334 ± 0.20	0.321***	2882	1.83 ± 0.31^b^	0.0034 ± 0.041	−0.005	1.34	0.404
Angus	19	56 ± 16	0.25 ± 0.15	0.341 ± 0.18	0.337*	1323	1.86 ± 0.25	0.0020 ± 0.029	0.015	1.25	0.258
Brahman	19	16 ± 5	0.14 ± 0.14	0.208 ± 0.19	0.203*	6228	1.61 ± 0.39	0.0061 ± 0.065	0.005	1.49	0.897

Het: heterozygosity; obs: observed; exp: expected; G: genomic relationship matrix; G diag: diagonal of G refereeing to inbreeding of the animal itself; G off-diag: average of off-diagonal element referring to relationship between animals; *A*_*R*_: allelic richness; *pA*_*R*_: private allelic richness

***P < 0.0001; *P < 0.01; ^†^< 0.05, t-test for differences between obs. and exp. heterozygosities

^a, b^: no significant difference between *A*_*R*_ means for indicated breeds

Further, we estimated the effective population size for five European taurine breeds as well as a Brahman population which were much lower than previously reported [17, 18]. Nevertheless, in comparative terms, the two island populations had the smallest effective population size within our study.

The relationship between population size and genetic variability has been extensively studied and with a reduction in population size it would be expected that the genetic variability also decreases and inbreeding increases, which is visible as a loss of heterozygosity [12, 19]. A first simplistic measure is to look at the number of markers that are fixed in a population, i.e. show no variability. Over all breeds in this study, 14,629 markers were fixed in at least one population. Within the Korean cattle breeds, the island populations had 2.5 to 3.0 times as many loci fixed compared to the Hanwoo, confirming a potential reduction in genetic variability between the Korean island and mainland populations (Table 1). Allelic richness (*A*_*R*_) estimates also confirmed a reduction in genetic variability for the two island populations (Table 1), which were only undercut by the Brown Swiss and Brahman populations of this study. However, observed heterozygosities were 0.303 and 0.306 for Jeju Black and Chikso, respectively, and 0.312 for mainland Hanwoo, which were significantly higher than estimated expected heterozygosity levels for these breeds (Table 1). Edea et al. [20] reported a 0.1 higher expected heterozygosity for Hanwoo cattle which might be a result of differing quality control filtering criteria or the use of a lower density genotyping platform, resulting in different estimates of heterozygosity. Suh et al. [16] found higher expected heterozygosity levels for brown Hanwoo and Chikso, however, their study was based on 30 microsatellite markers which present a different data basis.

Similarly to our observed increased heterozygosity levels compared to expected frequencies, estimated inbreeding coefficients indicated an excess of heterozygous genotypes for the two island populations (Table 1). Variation in inbreeding was similar between chromosomes and showed only stronger deviations from 0 for the Brown Swiss population which is most likely due to the small sample size (Additional file 1: Figure S1). Even though the indication for an excess in heterozygous loci is marginal, it might point towards an advantage and selection for over-dominant gene expressions [21]. Selection under environmental pressure can be observed in declining populations because survival or fecundity of heterozygous individuals is increased [22]. It was shown that Jeju Black are more resistant to theileria infections [23] - a piroplasms parasite which causes anorexia, fever, anaemia and icterus -, and Hanwoo cattle showed a higher resistance to the bovine papillomavirus compared to Holstein cattle [24]. Nevertheless, reports on different adaption due to environmental pressure within the Hanwoo varieties are sparse and do not allow for an in depth interpretation. Further, this interpretation warrants some caution as there is a possibility of gene flow between these populations which could also have led to the increased heterozygosity observed. Records for Chikso and Jeju Black are very sparse and in an attempt to preserve these populations, there may have been some undocumented crossing with brown Hanwoo at some point in time.

To get an indication about whether environmental selection pressure resulted in a higher variability for loci close to genes related to health and fertility traits, we performed a gene enrichment analysis in an area of one Mb in either direction of segregating markers that were unique for each Korean breed (93 markers for Chikso, 212 markers for Jeju Black, and 1104 markers for Hanwoo). In total, 1531 protein coding genes were found in Chikso, 2519 genes in Jeju Black, and 10,282 genes in Hanwoo cattle.

As expected from the larger number of genes for the Hanwoo breed, more gene sets were identified as significantly enriched and mostly just reflect the unbalance in the number of genes in the various processes. Thus, most genes were unsurprisingly involved in general processes such as regulation of transcription, proteolysis or cell adhesion (Table 2). Nevertheless, one gene set with 48 genes was associated to response to viruses and unique for Hanwoo cattle. Another gene set consisting of 19 genes was associated with defence response to bacteria and unique to Jeju Black cattle (Table 2). Further, small gene sets unique to Jeju Black and Chikso are involved in immune functions such as beta defensins [25, 26]. This provides some evidence that environmental selection pressure may have been exerted on these populations, and thus, possibly led to a higher heterozygosity. Further, 35 genes unique to the mainland Hanwoo were involved in glycerophospholipid metabolism (KEGG 00564) and 19 genes in fat digestion and absorption (KEGG 04975). These two pathways may be involved in intramuscular fat accumulation which is one of the main breeding goals for brown Hanwoo cattle. Follow-up studies would be required to verify these suggested genes for their involvement in breed differentiation.

**Table 2:** Biological process of significantly enriched gene sets in the three Korean cattle breeds

	# Genes	Biological Process	GO number	*P*-value*
Jeju Black	38	Homophilic cell adhesion	GO:0007156	<0.001
	57	Cell adhesion	GO:0007155	<0.001
	19	Defence response to bacteria	GO:0042742	0.003
Chikso	5	Amine metabolic process	GO:0009308	0.03
Hanwoo	374	G-protein coupled receptor	GO:0007186	<0.001
	41	Defence response	GO:0006952	<0.001
	200	Positive regulation of transcription from RNA polymerase II promoter	GO:0045944	<0.001
	131	Negative regulation of transcription from RNA polymerase II promoter	GO:0000122	0.001
	48	Response to virus	GO:0009615	0.004
	240	Proteolysis	GO:0006508	0.002
	119	Intracellular signal transduction	GO:0035556	0.01
	37	Response to oxidative stress	GO:0006979	0.04

*FDR corrected

**Fig. 1:**
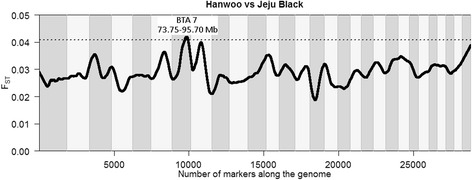
Marker-wise *F*_*ST*_ estimates between Hanwoo and Jeju Black. significance threshold (dotted line) is the average *F*_*ST*_ + 3 standard deviations

Locus differentiation based on *F*_*ST*_ values did not yield any significant differences between Hanwoo and Chikso, or Jeju Black and Chikso which confirms the closeness of these populations. One region on chromosome 7 (73,748,930–95,762,285 bp, Fig. 1), however, showed a signature of selection between Hanwoo and Jeju Black cattle which included uniquely segregating markers of the Hanwoo. This region has not been reported in other studies on selection signatures in Hanwoo cattle which might be due to the lower number of markers of this study compared to Porto-Neto et al. [27] or the novel intra-breed comparison of Hanwoo and Jeju Black. The identified region harbours 33 protein coding genes (Additional file 2: Table S1, UMD3.1), none of which can be immediately linked to the selection goals or environmental pressures of brown Hanwoo cattle.

**Fig. 2:**
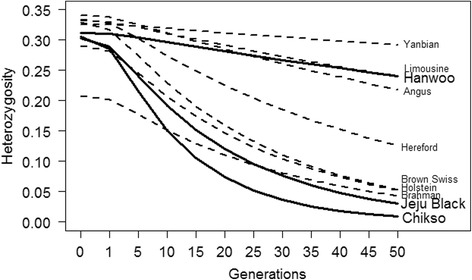
Estimated decay of heterozygosity in 10 cattle breeds over the next 50 generations. Solid lines: Korean cattle; dashed lines: other breeds

Even though the genetic variability of the two island populations is higher than expected and comparable to other cattle populations, a collapse of the island populations in the future could happen due to their small effective population sizes. Predicting the development of heterozygosity over the next 50 generations, without intervention through conservation programs, showed that the heterozygosity will drop drastically in the future and will halve in 10 to 15 generations (Fig. 2).

Counterintuitively given the population sizes, inbreeding cannot currently be observed in the island populations. However, an excess of heterozygous loci possibly due to environmental selection pressure together with the small effective population sizes warrant close monitoring of the populations to prevent inbreeding depression in the future.

### Differentiation between populations

The divergence between Yanbian and Hanwoo is very low and not significant (*F*_*ST*_ =0) despite the large geographic area and distance that these cattle inhabit. From a Korean production perspective Yanbian is of interest because it is an ideal population to provide insights into the effects of the breeding program on the genetic architecture of the Hanwoo (e.g. changes in *N*_*e*_, inbreeding, signatures of selection). Yanbian show higher levels of genetic diversity than Hanwoo which could be due to the intensive selection on the latter but this is potentially confounded with unaccounted crossing of Yanbian with other European breeds – Yanbian had lowest *F*_*ST*_ values with European breeds compared to the other Korean breeds (Table 3, Additional file 3: Figure S2 and Additional file 4: Figure S3).

**Table 3:** Pairwise estimates of *F*_*ST*_ after 100 bootstraps^a^ (upper diagonal) and *Nei’s D* (lower diagonal) between 10 cattle breeds

	HW	JB	CK	YB	HL	BS	LM	HF	AG	BR
HW	**0**	**0.016**	**0.014**	0^a^	0.131	0.114	0.085	0.138	0.106	0.253
JB	**0.024**	**0**	**0.036**	0.013	0.142	0.132	0.098	0.149	0.116	0.276
CK	**0.023**	**0.038**	**0**	0.015	0.147	0.140	0.105	0.154	0.121	0.287
YB	0.010	0.024	0.025	0	0.105	0.084	0.052	0.111	0.079	0.234
HL	0.093	0.106	0.108	0.080	0	0.111	0.082	0.118	0.083	0.305
BS	0.106	0.119	0.122	0.093	0.117	0	0.047	0.107	0.071	0.340
LM	0.071	0.084	0.087	0.055	0.081	0.088	0	0.075	0.042	0.281
HF	0.103	0.115	0.118	0.089	0.102	0.122	0.082	0	0.055	0.316
AG	0.080	0.092	0.095	0.066	0.077	0.099	0.060	0.065	0	0.274
BR	0.154	0.162	0.169	0.146	0.200	0.216	0.178	0.211	0.181	0

*HW* Hanwoo, *JB* Jeju Black, *CK* Chikso, *YB* Yanbian, *HL* Holstein, *BS* Brown Swiss, *LM* Limousine, *HF* Hereford, *AG* Angus, *BR* Brahman

^a^all *F*_*ST*_ estimates were significantly different from 0 (<0.0001) after 100 bootstraps apart from Hanwoo vs. Yanbian

Korean breeds are bold

**Fig. 3:**
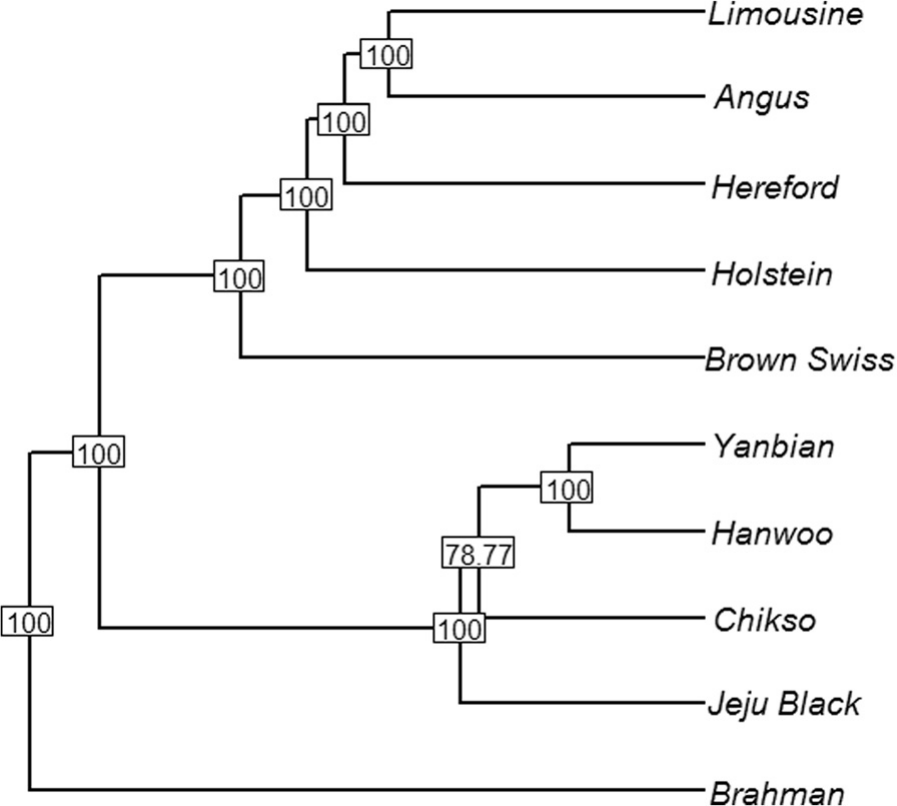
Phylogenetic tree of 10 cattle breeds based on allele frequencies of autosomes with 10,000 bootstrap replicas

The level of divergence between the two island populations was higher (*F*_*ST*_ 0.036) compared to the divergence to the mainland Hanwoo population at *F*_*ST*_ 0.016 and *F*_*ST*_ 0.014 (Table 3, Fig. 3, Additional file 3: Figure S2), fitting into a classical island model where the two island populations originated from the same mainland population and diverged in different directions over time. The admixture analysis confirmed the separation of the populations showing only spurious influences of indicine in all analysed taurine breeds (Additional file 4: Figure S3). The best number of ancestral populations was eight (K = 8), showing the smallest cross-validation standard error (0.544). At eight assumed ancestral populations, five groups were represented by the European taurine and the indicine outgroups (Additional file 4: Figure S3). The mainland Hanwoo and the Chinese Yanbian formed one group. Matching *F*_*ST*_, the Jeju Black is the first to split from the Brown Hanwoo to create a partial identity, whilst the Chikso is closely linked to Hanwoo with almost no Jeju Black *signal* (K = 7, Additional file 4: Figure S3). At K = 8, Chikso allelic frequencies are separable and there is hardly any overlap between the two island populations; but they both connect back to the mainland Hanwoo. This split confirms that the isolation of the island populations forced different trajectories of genetic change. Note that the Chikso population split into two subgroups; as previously described, the Chikso were sampled from two different provinces of South Korea and the two subgroups mirror this geographic separation. The brindle coloured population is probably quite heterogeneous between localities with restricted gene flow. Conservation measures should take into account that there are *pockets* of genetic diversity and improve the links between populations to contain further erosion of diversity due to drift or inbreeding.

The *F*_*ST*_ values within the Korean cattle populations ranged from 0.014 to 0.036 which is lower than the differences between well characterized European beef (0.042–0.075) or dairy breeds (0.111; Table 3). The molecular variance explained only 58.3 % of the variation between the Korean populations, whilst among European breeds, ~75.7 % of the variation was explained (Table 4). The divergence between the two island populations is close to the differences between closely related European breeds (0.036) but the differentiation from the mainland Hanwoo (0.016 and 0.014) is not as advanced as between cattle populations that have been classified as different breeds. Even though Jeju Black and Chikso are traditionally considered separate breeds from Hanwoo, the genetic evidence suggests that breed formation is still in process. Whether Jeju Black and Chikso will evolve to become fully distinct breeds or whether they will remain in Sisyphean evolution [3] will largely depend on future management decisions.

**Table 4:** Analysis of molecular variance in Korean, and European dairy and beef cattle populations

	Source of variance	Sum of square deviations	Mean of square deviations	Degrees of freedom	*F*-value	Variance components (%)
Korean	Between populations	0.205	0.103	2	5.15***	58.3
	Within populations	1.507	0.020	77		41.7
	Total	1.713	0.022	79		100
Europe	Between populations	1.032	0.258	4	10.32***	75.7
	Within populations	1.649	0.025	67		24.3
	Total	2.680	0.038	71		100

***p < 0.001: significance levels after 1000 permutations

Both Chikso and particularly the Jeju Black have a long association with the history of Korea – they serve as a *living link* to the past and to the cultural traditions of the country; hence, there is strong interest in preserving these populations for the future. The effective population sizes are small and whilst heterozygosity and inbreeding are currently not a major concern, forward projections of heterozygosity decay are quite troubling. Conservation efforts should focus on monitoring and maximizing diversity as well as tracking the overall robustness of the populations across time. There is a possibility that the Chikso is fragmented into isolates; a priority should be to improve gene flow between the subpopulations. A challenging question that will need to be addressed in the near future is whether to fully close these populations and work with the available diversity or introduce new variation from mainland Hanwoo. The relatively low differentiation at this point suggests that some level of introgression with Hanwoo and careful phenotypic selection for the population’s distinguishing traits would not compromise the integrity of the breeds but it may be politically infeasible to implement.

## Conclusion

The brown Hanwoo cattle have been subjected to artificial selection within a national breeding program aimed at meat quality for several decades. Two other varieties, the Jeju Black and the Chikso cattle, were excluded from the national breeding program and remained insulated from recent artificial selection advancements. This led to a decline in population sizes due to a lack of commercial interest in the breeds. Even though effective population sizes are small, there is currently little evidence of loss of genetic diversity or inbreeding. Nevertheless, forward estimates of heterozygosity project a rapid loss of diversity which justifies measures aimed at preserving the Jeju Black and Chikso population due to their historical and cultural relevance to Korea. The Jeju Black and Chikso varieties show some level of breed divergence from the mainland Hanwoo cattle, though distinctly less than between other well characterized cattle breeds. From a purely genetic perspective there is limited value in managing these populations independently; but given their high social value for Korea, a separate breeding program aimed at maximizing diversity and improving fitness is warranted.

## Methods

### Ethics statement

Sampling of the brown Hanwoo was carried out by veterinary practitioners in the Hanwoo Improvement Centre of the National Agricultural Cooperative Federation with the permission of the owners. The protocol was approved by the Committee on the Ethics of Animal Experiments of the National Institute of Animal Science (Permit Number: 2013–028). No ethics statement was required for the collection of other animal samples used in this study as data was provided by secondary sources.

### Sampling

Cattle were sampled from research stations on mainland Korea and Jeju Island. On Jeju Island, 20 Jeju Black cattle were sampled at the Subtropical Animal Experimental Station. Chikso cattle (*n* = 20) were collected from the Gyeonggi and Gyeonbuk provinces; and brown Hanwoo (*n* = 40) cattle were sampled from the Hanwoo Improvement Centre of the National Agricultural Cooperative Federation in Seosan (Chungnam province).

A further 39 animals from the Chinese Yanbian cattle breed were sampled from the Yanbian Prefecture in China. Finally, five originally European taurine breeds (*n* = 6 Brown Swiss, *n* = 19 Holstein, *n* = 15 Hereford, *n* = 19 Angus and *n* = 13 Limousine) and one indicine breed (*n* = 19 Brahman) were sampled from Australian populations and included in the analysis for comparison (Table 1). All animals were sampled at random and relationship statuses between animals of each population were confirmed using a genomic relationship matrix. The genomic relationship matrix was build according to Van Raden [28]. Missing genotypes were replaced by the average allele count across all animals.

### Genotyping

All animals were genotyped with the Illumina Bovine SNP 50 K Bead chip (Illumina, San Diego). Quality control was performed with *snpQC* [29] and the data was filtered based on call rates of markers and animals over 95 %, a median GC score for markers over 0.6, heterozygosity within three standard deviations from other SNPs and deviation from Hardy-Weinberg equilibrium for a cut-off *P*-value of 10^−16^. Markers on sex chromosomes or unmapped markers were excluded. Ascertainment bias of genotypes was checked by comparing results of segregating markers within populations versus results of markers segregating across populations. Bias was minor and therefore no further markers were excluded from the study. A total of 29,844 markers were used in the analyses.

### Data analyses

#### Genetic variability and admixture

The effective population size (*N*_*e*_) for each breed was estimated with the *LDNe* program [30]. Effective population sizes were estimated based on calculated *r*^*2*^ as linkage disequilibrium according to Hill [31] and Waples [32]. Due to constraints on the size of the input files, *N*_*e*_ was estimated per chromosome and then averaged across the entire genome. The mating system was chosen to be at random even though this is not fully realistic for livestock populations.

For purposes of conservation genetics, changes in heterozygosity over time were estimated as follows:

$$ {H}_t={H}_0{\left( 1- 1/\left( 2\kern0.5em {N}_e\right)\right)}^t $$

where *H*_*t*_ and *H*_*0*_ are the heterozygosity at time *t* and time zero, respectively [33, 34].

Allelic richness (*A*_*R*_) and private allelic richness (*pA*_*R*_) were estimated with the *HP-Rare v1.0* program [35], which includes differences in sample sizes to provide an unbiased estimate. Distribution of genetic variability between breeds, inbreeding and population differentiation were assessed with Wright [36] F-statistics (*F*_*IS*_ and *F*_*ST*_), estimated according to Weir and Cockerham [37], and Nei’s genetic distance as implemented in the *StAMPP* package in R [38, 39]. Significance test for differences in *F*_*ST*_ values were achieved by 100 bootstrapping replicas.

Euclidean distances and Ward’s clustering method as implemented in the *Ape* package in R were used to establish a phylogenetic tree [40, 41] based on allele frequencies. The phylogeny analysis was complemented with 10,000 bootstrapping replicas on the entire marker data with random replacements. Further, principal components based on a genomic relationship matrix [28] were assessed, and an analysis of molecular variance (AMOVA [42]) carried out to establish within and between population variation.

Finally, we used ADMIXTURE 1.23 [43] to predict ancestral populations and estimate breed proportions. The best number of ancestral populations (K) was inferred through cross-validation of 1 to 10 assumed populations.

#### Gene enrichment

The numbers of fixed and segregating markers were assessed and the overlap of markers between breeds calculated. A gene enrichment analysis for segregating markers that were unique for each of the Korean breeds was carried out. Genes including a direct marker or genes within one Mb in either direction of a marker (to minimize the possibility of recombination between marker and causal mutation) were regarded as adaptive variation whereas all other markers were regarded as neutral variation. The *GeneCodis* program (release 3) [44–46] was used to filter whether gene groups with similar functions occurred more frequently than expected between breeds. A Chi^2^ test was used to compute *P*-values which were corrected for multiple testing through false-discovery rate [47].

## Availability of supporting data

Datasets will be made available upon acceptance of this paper and before publication. Genotypic data of the Korean cattle breeds are deposited with Dryad: http://dx.doi.org/10.5061/dryad.p2b3b.

## Additional files

Additional file 1: Figure S1. Inbreeding coefficient (*F*_*IS*_) per chromosome for 10 cattle breeds. (TIF 238 kb) Additional file 2: Table S1. Genes on chomosome 7 within the region of the selection signature (*F*_*ST*_) for Hanwoo vs. Jeju Black cattle. (PDF 43 kb) Additional file 3: Figure S2. Plots of principal components based on genomic relationships of 3 Korean and 7 outgroup cattle breeds. PC1 represents the separation between taurine and indicene breeds PC2 represents the separation between the European and Asian taurine breeds. (TIFF 34 kb) Additional file 4: Figure S3. Estimated breed proportions for 2–9 assumed ancestral populations (ADMIXTURE 1.23). K = 8 had the smallest cross-validation standard error. (TIF 369 kb)
