# Supplementary figures and images for: Towards breed formation by island model divergence in Korean cattle
Source: BMC Evol Biol. 2015 Dec 18;15:284. doi: 10.1186/s12862-015-0563-2 (PMC4683938; doi:10.1186/s12862-015-0563-2)

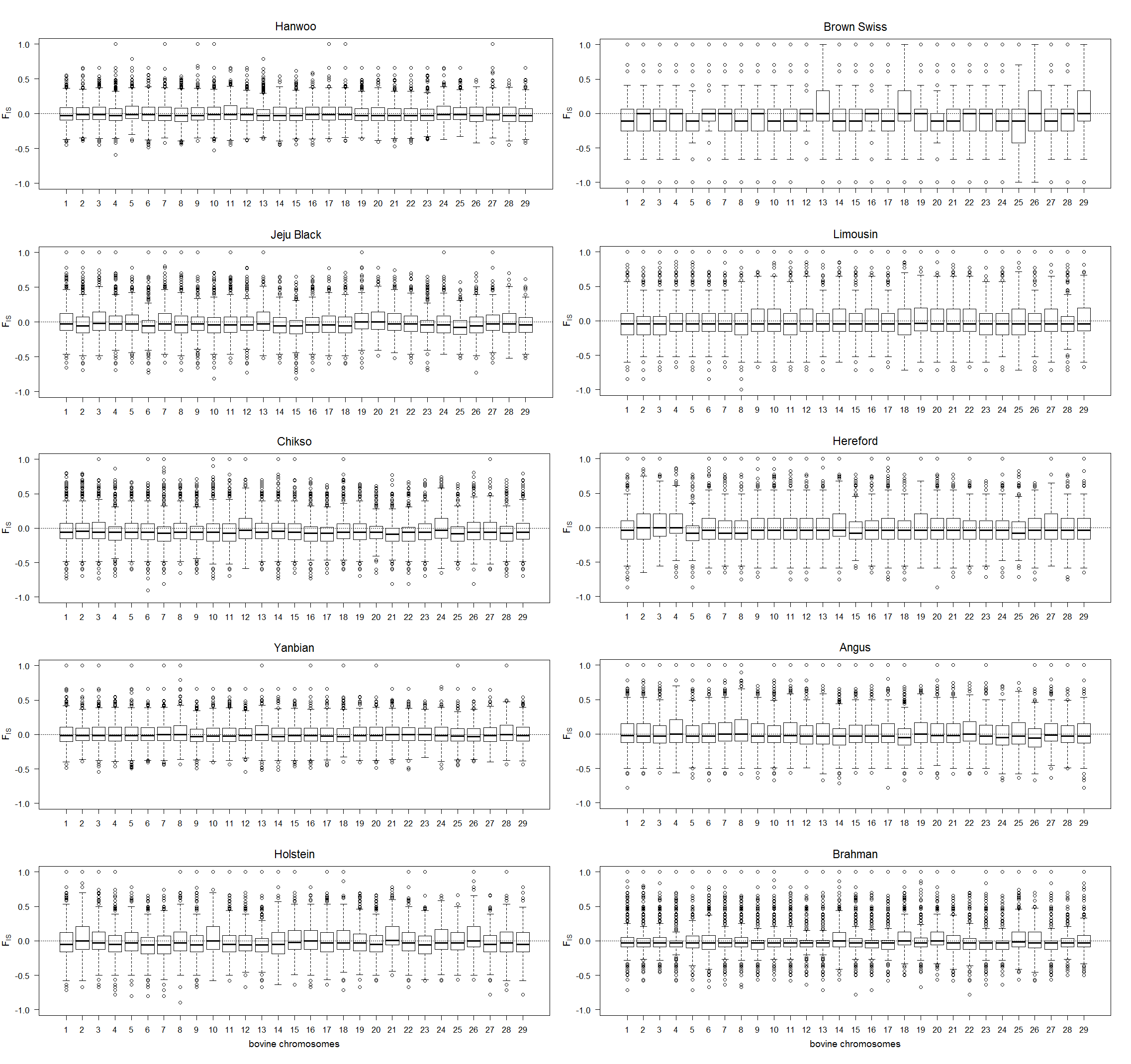

Supplement: Additional file 1: Figure S1. — Inbreeding coefficient (F IS) per chromosome for 10 cattle breeds. (TIF 238 kb) [file 12862_2015_563_MOESM1_ESM.tif]

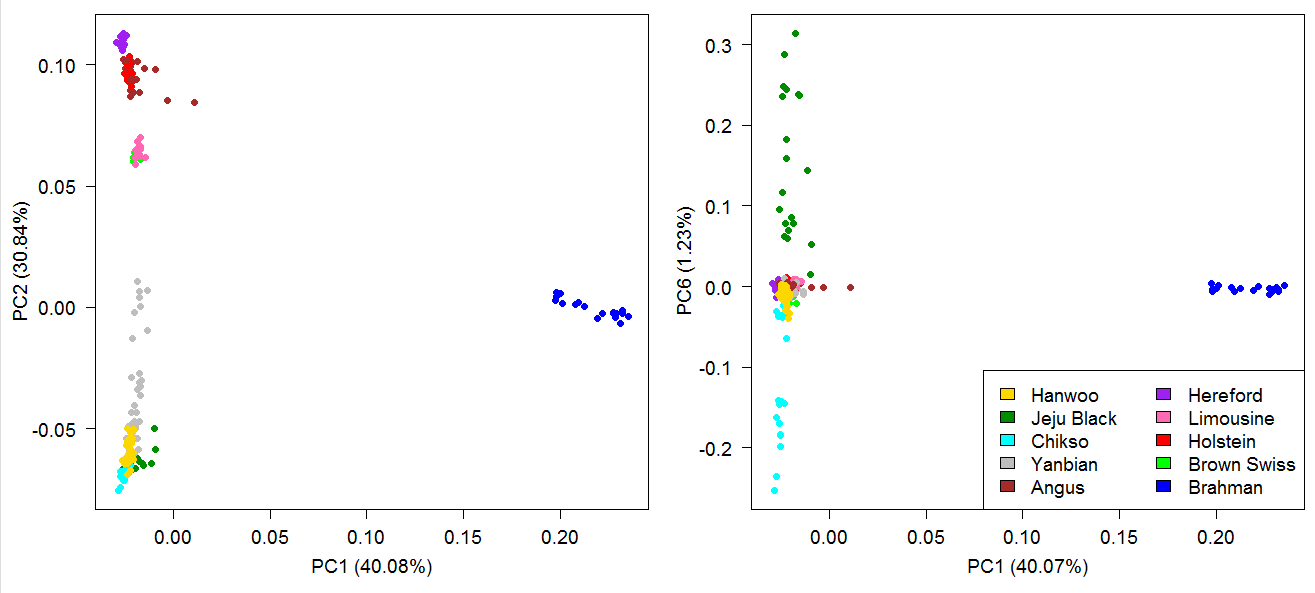

Supplement: Additional file 3: Figure S2. — Plots of principal components based on genomic relationships of 3 Korean and 7 outgroup cattle breeds. PC1 represents the separation between taurine and indicene breeds PC2 represents the separation between the European and Asian taurine breeds. (TIFF 34 kb) [file 12862_2015_563_MOESM3_ESM.tiff]

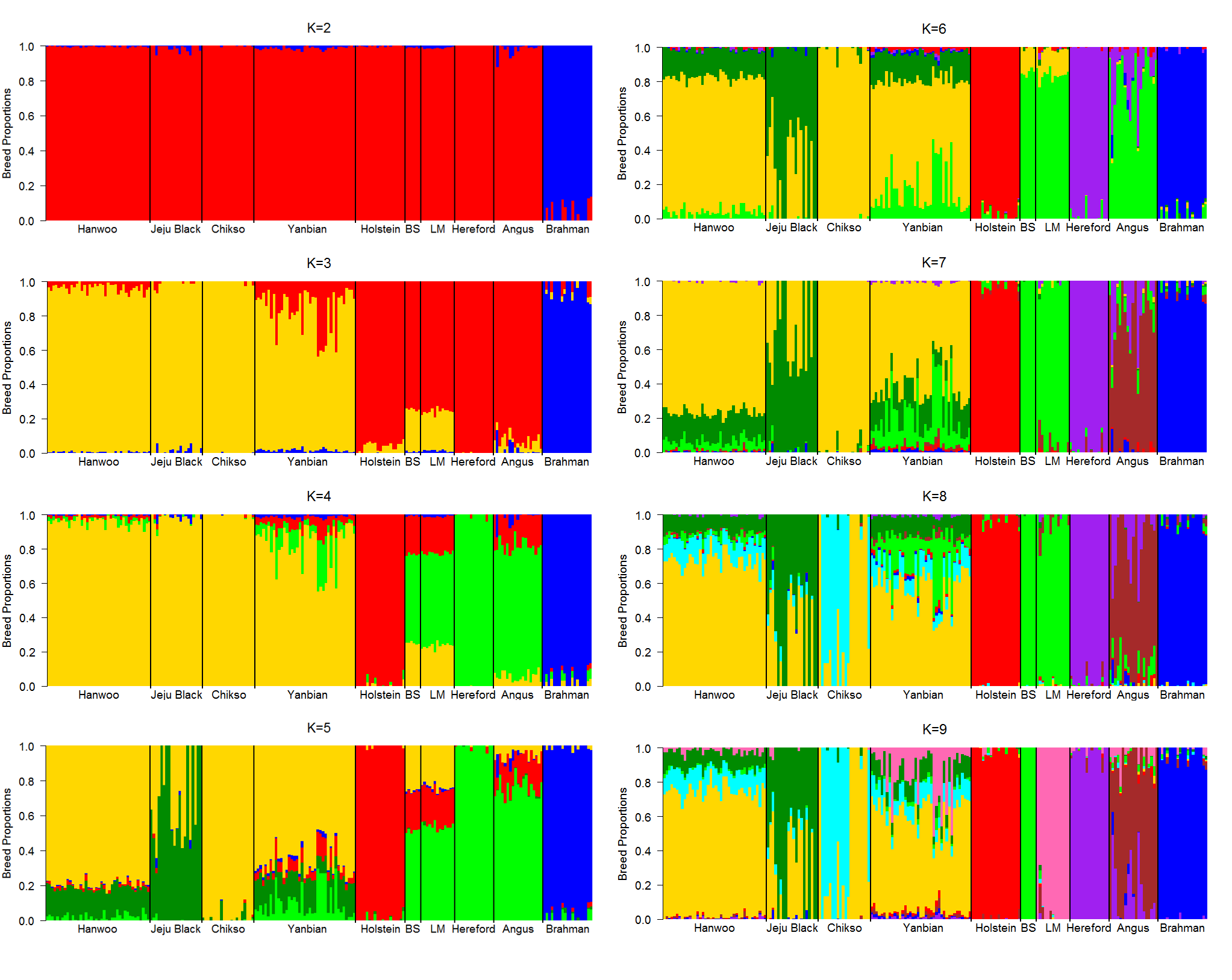

Supplement: Additional file 4: Figure S3. — Estimated breed proportions for 2–9 assumed ancestral populations (ADMIXTURE 1.23). K = 8 had the smallest cross-validation standard error. (TIF 369 kb) [file 12862_2015_563_MOESM4_ESM.tif]
