# Supplementary material for: Towards breed formation by island model divergence in Korean cattle
Source: BMC Evol Biol. 2015 Dec 18;15:284. doi: 10.1186/s12862-015-0563-2 (PMC4683938; doi:10.1186/s12862-015-0563-2)
Supplement: Additional file 2: Table S1. — Genes on chomosome 7 within the region of the selection signature (F ST) for Hanwoo vs. Jeju Black cattle. (PDF 43 kb) [file 12862_2015_563_MOESM2_ESM.pdf]

Supplementary Table 1. Genes* on chromosome 7 within the region of the selection signature (F_{ST}) for Hanwoo vs. Jeju Black cattle

Gene	Start Position	End Position	Description
ATP10B	74,334,045	74,472,502	Uncharacterized protein
NA	75,043,589	75,043,969	Uncharacterized protein
GABRB2	75,103,433	75,393,342	gamma-aminobutyric acid receptor subunit beta-2 precursor
BT.39107	75,522,540	75,539,456	gamma-aminobutyric acid receptor subunit alpha-6
GABRA1	75,703,666	75,759,741	gamma-aminobutyric acid receptor subunit alpha-1
GABRG2	75,895,256	76,022,776	gamma-aminobutyric acid receptor subunit gamma-2 precursor
CCNG1	77,249,997	77,257,261	Cyclin-G1
NUDCD2	77,264,728	77,270,660	nudC domain-containing protein 2
BT.12993	77,271,070	77,303,644	Uncharacterized protein
MAT2B	77,321,513	77,337,009	Methionine adenosyltransferase 2 subunit beta
GNG5P2	80,188,341	80,188,849	Guanine nucleotide-binding protein subunit gamma
BT.90094	83,040,940	83,227,242	DNA mismatch repair protein Msh3
RASGRF2	83,362,788	83,546,393	Uncharacterized protein
CKMT2	83,554,315	83,579,633	creatine kinase S-type, mitochondrial precursor
BT.61596	83,599,489	83,607,997	zinc finger CCHC domain-containing protein 9
ACOT12	83,618,466	83,658,727	acyl-coenzyme A thioesterase 12
SSBP2	83,706,304	84,016,324	single-stranded DNA-binding protein 2
MGC138057	84,242,450	84,505,756	ubiquitin-like-conjugating enzyme ATG10
RPS23	84,523,780	84,525,639	40S ribosomal protein S23
EDIL3	86,158,700	86,638,968	Uncharacterized protein
COX7C	88,648,457	88,650,528	Cytochrome c oxidase subunit 7C, mitochondrial
BT.102247	89,281,002	89,391,377	ras GTPase-activating protein 1
CCNH	89,393,228	89,423,692	cyclin-H
TMEM161B	90,097,694	90,176,089	transmembrane protein 161B
MEF2C	90,616,543	90,784,162	Myocyte-specific enhancer factor 2C
NA	92,280,134	92,281,108	Uncharacterized protein
CETN3	92,290,239	92,307,723	centrin-3
